# A Survey of UK Healthcare Workers’ Attitudes on Volunteering to Help with the Ebola Outbreak in West Africa

**DOI:** 10.1371/journal.pone.0120013

**Published:** 2015-03-11

**Authors:** Lance Turtle, Fiona McGill, Judy Bettridge, Claire Matata, Rob Christley, Tom Solomon

**Affiliations:** 1 Institute of Infection and Global Health, University of Liverpool, Ronald Ross Building, Liverpool, L69 7BE, United Kingdom; 2 NIHR Health Protection Research Unit, University of Liverpool, Ronald Ross Building, Liverpool, L69 7BE, United Kingdom; 3 Walton Centre NHS Foundation Trust, Lower Lane, Liverpool, L9 7LJ, United Kingdom; University of Massachusetts Medical School, UNITED STATES

## Abstract

**Objective:**

To understand the barriers and enablers for UK healthcare workers who are considering going to work in the current Ebola outbreak in West Africa, but have not yet volunteered.

**Design:**

After focus group discussions, and a pilot questionnaire, an anonymous survey was conducted using SurveyMonkey to determine whether people had considered going to West Africa, what factors might make them more or less likely to volunteer, and whether any of these were modifiable factors.

**Participants:**

The survey was publicised among doctors, nurses, laboratory staff and allied health professionals. 3109 people answered the survey, of whom 472 (15%) were considering going to work in the epidemic but had not yet volunteered. 1791 (57.6%) had not considered going, 704 (22.6%) had considered going but decided not to, 53 (1.7%) had volunteered to go and 14 (0.45%) had already been and worked in the epidemic.

**Results:**

For those considering going to West Africa, the most important factor preventing them from volunteering was a lack of information to help them decide; fear of getting Ebola and partners’ concerns came next. Uncertainty about their potential role, current work commitments and inability to get agreement from their employer were also important barriers, whereas clarity over training would be an important enabler. In contrast, for those who were not considering going, or who had decided against going, family considerations and partner concerns were the most important factors.

**Conclusions:**

More UK healthcare workers would volunteer to help tackle Ebola in West Africa if there was better information available, including clarity about roles, cover arrangements, and training. This could be achieved with a well-publicised high quality portal of reliable information.

## Introduction

On 21st March 2014, the World Health Organisation was officially notified of an outbreak of Ebola virus disease due to *Zaire ebolavirus* in Guinea, Liberia and Sierra Leone. The outbreak was declared a “public health emergency of international concern” on 8th August 2014 [1]. As of January 9^th^ 2015, a total of 21086 cases (13376 laboratory confirmed) and 8289 deaths have been reported [2]. The epidemic is currently doubling approximately every 4 weeks and the case fatality rate, when based on the most accurate available information, is around 70 percent [1]. Small numbers of cases have occurred in Nigeria, Senegal and Mali. In late September the outbreak became transcontinental with the importation of a previously subclinical case to Texas, USA from Liberia [3]. Onward transmission occurred in the healthcare facility in Texas [3], and also occurred in Spain, following the repatriation of an infected healthcare worker [4]; more countries in West Africa may also be at risk of Ebola [5]. This is an exceptional situation with the potential for spread to almost any country in the world [6].

The global response to the outbreak has been slow. As early as April 2014 Médecins Sans Frontières (MSF) warned that this outbreak was “unprecedented” [7]. MSF has criticised the speed of response on several occasions [8,9] and on 5th September 2014, the number of deaths reported to WHO in this outbreak surpassed those in all other known outbreaks combined [1,10–15]. In October 2014 Oxfam suggested that the world had only two months to get the epidemic under control [16].

Tackling the current Ebola virus outbreak requires a global response in terms of money, infrastructure and people. On the 21st October 2014 MSF had only 270 international staff and 3018 local staff working in Guinea, Liberia and Sierra Leone [17]. The World Bank have called for at least 5000 more medical and support staff [18]. In addition to the World Bank, organisations such as MSF, WHO and UNICEF have called for more qualified staff to help [19]. In the UK, approximately 1000 healthcare workers have so far volunteered to go to West Africa to help in the response [20]. Many more have considered going, but not yet volunteered.

There are likely to be many factors that influence a person’s decision regarding whether or not to volunteer in a situation like this. We wanted to understand what these factors are, and in particular whether any of them might be amenable to intervention or influence. Knowledge of the relative contributions of the different enablers and barriers might guide UK policymakers as to what is needed to ensure more healthcare workers volunteer to help control the Ebola outbreak. Therefore, we conducted a survey of UK health professionals to understand their attitudes towards going to work in the Ebola epidemic in West Africa.

## Methods

To understand what some of the potential barriers and enablers might be that influence the decision of healthcare workers over volunteering to go to West Africa we examined social media, blogs and online comments (see S1 File for information sources). We also conducted small focus group discussions of healthcare workers. Based on these we produced a draft questionnaire which we piloted on a small number of different healthcare workers before modifying into the final version (S3 File). Briefly, the questionnaire asked whether respondents had considered going to work in the Ebola outbreak and what decision they had come to. Two questions investigated what the barriers and enabling factors were according to a 5 point Likert scale from “strongly agree” to “strongly disagree.” The fourth question concerned where respondents got their information on Ebola from, and subsequent questions gathered demographic information such as profession, age, sex and level of experience. Free text boxes were included to pick up any other concepts not initially identified in the questionnaire and to enable participants to elaborate on their responses. We used the web based Surveymonkey to create and distribute the questionnaire.

### Ethics statement

The questionnaire and study protocol were approved by the University of Liverpool research ethics committee (RETH 000774).

The survey went live on Wednesday 15th October 2014 and was disseminated using multiple means including various professional colleges, societies, training bodies, letters to the BMJ [21] and the nursing press. A list of the organisations that disseminated the questionnaire is shown in the S3 File. It was also advertised informally by word of mouth and using social media. The survey, which can be found at www.surveymonkey/s/HPRUebola, was entirely anonymous. Initial data were reviewed after one week, the free text comments were analysed and recurrent concepts identified (1450 respondents). These initial responses were used to modify the questionnaire through inclusion of 2 additional barriers and 4 enabling factors (S4 File). The revised questionnaire went live on Wednesday 22^nd^ October 2014. Responses were downloaded as comma separated values at 9:20pm on 4^th^ November 2014 (S1 Dataset).

Analysis was conducted using R software version 2.15.3 (R core team 2013). Responses were analysed descriptively and proportions of respondents giving certain answers were calculated. In order to rank the relative importance of barriers and enablers, values were assigned to responses on the Likert scale (“Strongly agree”, +2; “Agree”, +1”; “Neither disagree nor agree”, 0; “Disagree”, -1; “Strongly disagree” -2) and the total score for each barrier or enabler was calculated. We wished to explore whether demographic or other factors accounted for any responses observed. We were also interested in identifying whether particular barriers may cluster together. This is important because these barriers would all need to be addressed to affect an individual’s decision; barriers that did not cluster could be dealt with in isolation. In order to address these questions simultaneously, we used redundancy analysis, a form of multivariate analysis that combines a principal component analysis, to identify clusters, with regression to identify significant explanatory variables; this analysis used the R package “vegan” [22], according to the methods described by Borcard [23]. A matrix of explanatory variables was constructed for the redundancy analysis, on which the response to each barrier was regressed. A forward selection process was used to select significant variables which explained the greatest proportion of variance in the response data, and permutation tests used to test significance of RDA axes. Triplots were produced according to correlations between variables (scaling 2 in the vegan package).

## Results

A total of 3109 people completed the survey between 15^th^ October and 4^th^ November 2014. Two thousand and ninety eight (68%) respondents were doctors, 674 (22%) were nurses and the remainder were a mixture of armed forces health professionals, paramedics, pharmacists and a wide range of other allied health professionals (3% did not give their profession). The largest group of respondents, 943 (31%), worked in acute specialties such as acute medicine, emergency medicine and intensive care (Table 1). Medical specialties came next with 728 (24%) respondents, followed by others, including primary care, infection specialties, paediatrics, surgery and obstetrics & gynaecology. Respondents were generally experienced, 77% having more than 5 years of experience since their primary health care qualification and 55% with more than 10 years. Fifty-one percent of those answering had children or other dependents at home.

**Table 1:** Demographic information for all the respondents who completed the survey, divided according to the answer to whether they have considered going to West Africa to help in the current Ebola virus epidemic.

		*Considering*		*Not Considered*		*Decided Against*		*Volunteered*		*Already Been*		*No Answer*		*Total*	
		n = 472		n = 1791		n = 704		n = 53		n = 14		n = 75		n = 3109	
**Age**	Under 20	1	(0.2)	2	(0.1)	1	(0.1)	0	(0)	0	(0)	0	(0)	4	(0.1)
	20–25	23	(4.9)	34	(1.9)	22	(3.1)	1	(1.9)	0	(0)	1	(1.3)	81	(2.6)
	26–35	205	(43.4)	643	(35.9)	277	(39.3)	17	(32.1)	5	(35.7)	16	(21.3)	1163	(37.4)
	36–45	100	(21.2)	525	(29.3)	185	(26.3)	21	(39.6)	3	(21.4)	19	(25.3)	853	(27.4)
	46–65	118	(25)	496	(27.7)	187	(26.6)	11	(20.8)	4	(28.6)	32	(42.7)	848	(27.3)
	over 65	11	(2.3)	26	(1.5)	10	(1.4)	1	(1.9)	1	(7.1)	2	(2.7)	51	(1.6)
	No answer	14	(3)	65	(3.6)	22	(3.1)	2	(3.8)	1	(7.1)	5	(6.7)	109	(3.5)
**Sex**	Female	265	(56.1)	1104	(61.6)	390	(55.4)	24	(45.3)	6	(42.9)	40	(53.3)	1829	(58.8)
	Male	185	(39.2)	619	(34.6)	284	(40.3)	27	(50.9)	7	(50)	29	(38.7)	1151	(37)
	No answer	22	(4.7)	68	(3.8)	30	(4.3)	2	(3.8)	1	(7.1)	6	(8)	129	(4.1)
**Children**	No	333	(70.6)	702	(39.2)	323	(45.9)	37	(69.8)	9	(64.3)	30	(40)	1434	(46.1)
	Yes	117	(24.8)	1001	(55.9)	351	(49.9)	13	(24.5)	4	(28.6)	37	(49.3)	1523	(49)
	No answer	22	(4.7)	88	(4.9)	30	(4.3)	3	(5.7)	1	(7.1)	8	(10.7)	152	(4.9)
**Origin**	UK	389	(82.4)	1506	(84.1)	588	(83.5)	38	(71.7)	10	(71.4)	61	(81.3)	2592	(83.4)
	EU	29	(6.1)	90	(5)	38	(5.4)	7	(13.2)	1	(7.1)	1	(1.3)	166	(5.3)
	West Africa	6	(1.3)	9	(0.5)	7	(1)	0	(0)	0	(0)	0	(0)	22	(0.7)
	Other Sub-Saharan Africa	3	(0.6)	14	(0.8)	13	(1.8)	1	(1.9)	0	(0)	1	(1.3)	32	(1)
	Other nationality	21	(4.4)	90	(5)	31	(4.4)	4	(7.5)	2	(14.3)	3	(4)	151	(4.9)
	No answer	24	(5.1)	82	(4.6)	27	(3.8)	3	(5.7)	1	(7.1)	9	(12)	146	(4.7)
**Profession**	Doctor	312	(66.1)	1179	(65.8)	553	(78.6)	43	(81.1)	11	(78.6)	43	(57.3)	2141	(68.9)
	Nurse	118	(25)	446	(24.9)	103	(14.6)	5	(9.4)	2	(14.3)	21	(28)	695	(22.4)
	Allied health professional	14	(3)	65	(3.6)	12	(1.7)	2	(3.8)	0	(0)	3	(4)	96	(3.1)
	Biomedical scientist	11	(2.3)	20	(1.1)	11	(1.6)	1	(1.9)	0	(0)	0	(0)	43	(1.4)
	Admin	0	(0)	8	(0.4)	0	(0)	0	(0)	0	(0)	0	(0)	8	(0.3)
	Research	2	(0.4)	7	(0.4)	1	(0.1)	0	(0)	0	(0)	1	(1.3)	11	(0.4)
	Other	2	(0.4)	6	(0.3)	2	(0.3)	0	(0)	0	(0)	0	(0)	10	(0.3)
	No answer	13	(2.8)	60	(3.4)	22	(3.1)	2	(3.8)	1	(7.1)	7	(9.3)	105	(3.4)
**Specialty**	Emergency medicine, acute medicine, intensive care & anaesthesia	144	(30.5)	574	(32)	201	(28.6)	17	(32.1)	2	(14.3)	26	(34.7)	964	(31)
	Infection specialty	72	(15.3)	103	(5.8)	110	(15.6)	14	(26.4)	5	(35.7	6	(8)	310	(10)
	Other medical specialty	76	(16.1)	515	(28.8)	160	(22.7)	8	(15.1)	0	(0)	14	(18.7)	773	(24.9)
	Community/primary care	61	(12.9)	162	(9)	92	(13.1)	5	(9.4)	1	(0.1)	9	(12)	330	(10.6)
	Paediatric specialty	37	(7.8)	115	(6.4)	44	(6.3)	2	(3.8)	3	(21.4)	5	(6.7)	206	(6.6)
	Surgical specialty	13	(2.8)	54	(3)	21	(3)	0	(0)	0	(0)	5	(6.7)	93	(3)
	Obstetrics & gynaecology	12	(2.5)	25	(1.4)	9	(1.3)	0	(0)	0	(0)	1	(1.3)	47	(1.5)
	Other	39	(8.3)	177	(9.9)	44	(6.3)	4	(7.5)	2	(14.3)	4	(5.3)	270	(8.7)
	No answer	18	(3.8)	66	(3.7)	23	(3.3)	3	(5.7)	1	(7.1)	5	(6.7)	116	(3.7)

Data are numbers (percent).

Four hundred and seventy-two (15%) respondents were considering going to West Africa to help with the Ebola virus epidemic, but had not yet volunteered (*“Considering”*); 1791 (58%) had not considered going (*“Not Considered”*); 704 (23%) had considered it and decided not to go (*“Decided Against”*); 53 (1.7%) had made definite plans to go and 14 (0.4%) had already been to West Africa to help in the outbreak.

**Fig 1:**
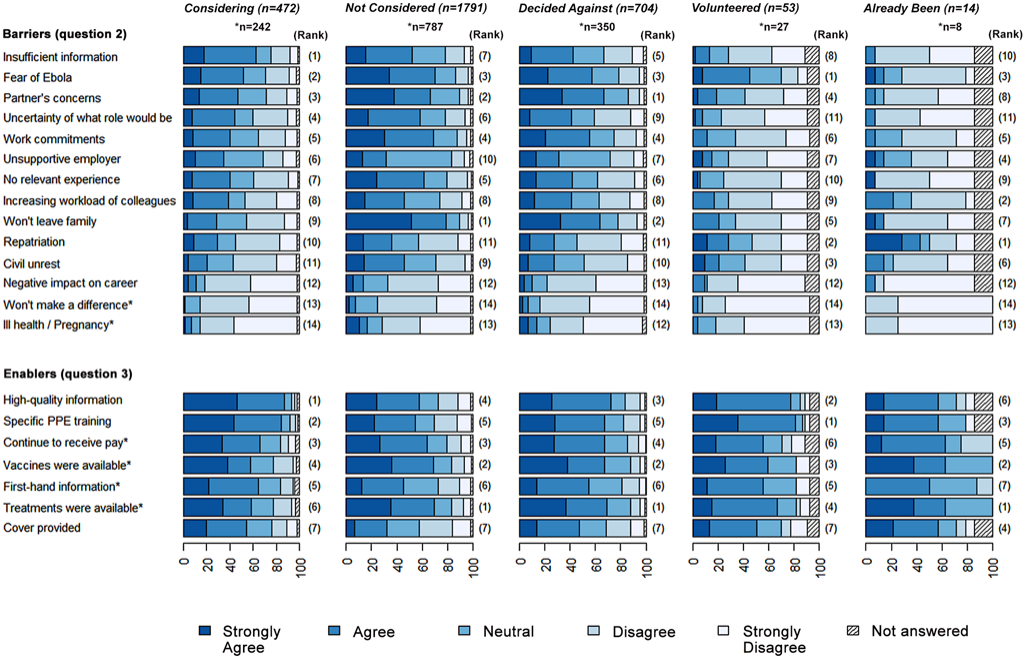
Barriers and enablers to going to West Africa to help with the Ebola outbreak for four groups of respondents. The importance of each issue is indicated on a 5 point Likert scale from strongly disagree to strongly agree, for those who were considering going but had not yet decided (*“Considering”*); those who had not considered going (*“Not Considered”*); those who had considered it and decided not to go (*“Decided Against”*); those who had volunteered and were waiting to go (“*Volunteered*”), and those who had already been (“*Already Been*”). Issues marked * were introduced in the second version of the questionnaire from 22^nd^ October onwards (1450 responses). Data are the percentage of respondents giving the answers indicated; and the rank is indicated showing how important that issue was for that group. The values from which the figure is derived are given in S1 and S2 Tables.

Our analysis focussed on the 472 people in the *Considering* group, because this is the group who may be willing to go to West Africa. For people in this group, the most important barrier identified for not yet having volunteered was insufficient information to reach a decision (Fig. 1). Some of the areas of what information is required can be summed up in this quote:

“Lack of information is my main barrier. I have no idea whether my skill-set would be useful there, if I am needed there, how to go about joining the efforts, or how to negotiate the time off with my trust. Any information would be greatly appreciated.”

The main areas where information could be targeted are shown in Table 2. In particular, although there is a dedicated website where NHS employees can express their interest (http://uk-med.humanities.manchester.ac.uk/), it is clear from these responses that this website is not widely known by many people (including those who would like to go and help). Additionally the information people are seeking is not available, either on that website or elsewhere. People would appear to welcome a more direct appeal for help. Amongst doctors in training in particular there was a need for clarity on how it would affect their training programmes. There were also many comments regarding the lack of information concerning exactly what skills or experience would be useful. Finally the main area of clarity required is getting timely responses from the organisations sending people out to help. Areas of lesser concern that nevertheless caused respondents to comment were the risk of contracting Ebola and medical evacuation.

**Table 2:** Areas of information required by healthcare workers considering going to work in West Africa.

Area of information required	Example responses
**Exactly how people can volunteer**	*“I am frustrated*. *I don’t know how to get out there*. *I want to go”*
	*“I really want to go*, *but I don’t know how and with which organisation*.*”*
	*“I would love to go and help*, *but am unsure of how to go about doing it”*
	*“I would be keen to go but I don’t know how I would get involved”*
	*“Don’t know how or to whom I can talk to to make this happen”*
	*“I would love to go and help out in West Africa but I have no idea how I would go about volunteering”*
	*“I haven’t come across a ‘one-stop’ site for info re potential NHS volunteers……….is there one I’ve missed*?*”*
	*“Need a simple and well publicised sign up procedure”*
	*“I would have liked the opportunity to attend an event for potential volunteers to get an idea of whether I could do something useful there without any commitment at this stage”*
**Need for information to be more directly disseminated to front line healthcare workers**	*“I would have liked the opportunity to attend an event for potential volunteers to get an idea of whether I could do something useful there without any commitment at this stage”*
	*“If you need NHS staff to volunteer to help with the Ebola outbreak*, *you need to approach staff more directly*. *I cannot remember having any email requesting that I consider it*.*”*
	*“Until I read the letters in the BMJ I wasn’t aware that volunteers were being proactively recruited”*
	*“I have not been made aware of any official drive for UK doctors to travel to the outbreak*, *but would certainly be interested in doing so*. *Do get in touch*.*”*
	*“Bring information to us*, *i*.*e*. *a recruitment drive within the NHS to give us all the info”*
	*“I personally haven’t been aware of any campaigns/information given to health care workers asking for volunteers*. *Only aware of it due to media coverage”*
**Need for responses from organisations**	*“I have registered interest but have not heard any further details”*
	*“I applied when NHS workers were first asked to volunteer………I never heard back*. *When I emailed to find out what had happened*, *I did not receive a reply*.*”*
	*“Have applied and arranged for leave from work but have not as yet had a response”*
**What skills and experience are actually needed**	*“I would love to go and help*, *but am unsure……if I have useful skills”*
	*“If I felt there was clear information about what roles were needed filled in West Africa*, *I would more seriously consider if I could go*.*”*
	*“I currently feel unsure how I could help………If I felt there was a useful task I could undertake in response to the outbreak*, *I would then more seriously weigh up the risks…*.*”*
	*“I do not know if my clinical skills would be of any use*!*”*
	*“As a junior doctor I want to help but don’t know if I would be eligible or useful*. *It would be really helpful if UKIEMR/other organisations published a list of necessary/desirable qualities*.*”*
	*“There is particularly poor quality information available on what role Public Health specialists could fulfil*, *including academic epidemiologists*, *analysts and so on*.*”*
	*“More information is needed on what skills are needed and what training will be provided*.*”*
**Will employers/training directors release staff**	*“I would love to go and help*, *but am unsure if my current Trust would even release me from my NTN [national training number]*.*”*
	*“I think I would be willing to travel out to help if I got more information about it*, *and if there was some way that deaneries would let me out*.*”*
	*“[I have] no information about if it’s possible to go and to leave a training post*.*”*
	*“I do not feel my current employer has offered any information or guidance for anyone of my level wishing to offer assistance a part of the Ebola response and there has been no information as to how our training would be affected”*
	*“There doesn’t seem to have been much encouragement from bodies such as the RCN*, *NMC*, *or the NHS itself regarding this*. *It might help if some kind of structured programme was promoted*, *offering more information about what skills and experience are needed*, *what exactly would be expected*, *what sort of timescale would be involved*, *and assurance from employers that salary*, *jobs and pensions would be protected*.*”*
**Risk of infection**	*“Although I am potentially willing to help there is no clear information about current infection rates amongst staff volunteering*, *and about methods of keeping safe…… I need this information to weigh the risks to myself and family against the urge to assist*. *I do not know where this information could be found*.*”*
**Need for information regarding evacuation**	*“I contacted NGOs working in the West Africa and I once agreed to work in the field with one of these NGOs*. *However*, *I could not obtain appropriate information on risk management such as emergency evacuation; thus my employer did not agree for me to work in the west Africa*.*”*
	*“… there is no clear guidance about what would happen if one of us contracted the virus*.*”*
	*“The main concern is the uncertainty regarding medical evacuation”*
	*“Once…..the rules for medical evacuation are defined I would most likely be willing to go…”*
	*“I would find it easier to decide if I had more information re options for participating*, *such as job descriptions*, *location*, *set-up*, *training*, *medical insurance*, *medical evacuation if needed*.*”*

The next two barriers were the fear of getting Ebola, and a partner’s concerns about them going. Further important issues for the *Considering* group included uncertainty around what their role would be in the epidemic, work commitments at home and not being able to get sufficient time off from their employer. The free text comments exemplified many of these issues:

“Lack of information is my main barrier. I have no idea whether my skill-set would be useful there, if I am needed there, how to go about joining the efforts, or how to negotiate the time off with my trust.” (Male, gastroenterology registrar.)

“As a junior doctor I want to help but don’t know if I would be eligible or useful. It would be really helpful if UKIEMR/other organisations published a list of necessary/desirable qualities.”(Male, F2, acute medicine,)

“My host trust refuses to let me go due to significant staff shortages of middle grade doctors of appropriate training to fill my place, and also currently they do not have the funds to replace me for 3 months. I offered to take unpaid leave and for my salary to be used by the Trust to fund a locum”. (Female, emergency medicine registrar.)

The barriers for people in the *Considering* group were very different to those in the *Not Considered* and *Decided Against* groups; for these two groups, many of the issues were similar. Thus family commitments and partners concerns were the two most important issues, and insufficient information was much less important (Fig. 1). Of note, nurses in the group that *Decided against* going to work in the epidemic were more likely that other groups of respondents to answer that their employer prevented them from volunteering.

The demographics of the groups also differed; 43% of those considering going were in the 26–35 age group, compared with 36% of those not considering going, and 39% of those who had decided against going (Table 1; χ^2^ test p = 0.002). Those considering going were also less likely to have children (χ^2^ test p < 0.0001). They also less frequently “Agreed” or “Strongly Agreed” with the questions that emphasised the barriers to going, for example they were significantly less afraid of contracting Ebola (χ^2^ test p < 0.0001). Interestingly, fear of Ebola was associated with getting information predominantly from the media in the group considering going but who had not yet volunteered. Conversely, across all respondents to the survey, getting information predominantly from the medical literature (irrespective of how much information was from the media) was associated with a roughly fourfold decrease in fear of contracting Ebola (χ^2^ test p < 0.0001).

For those considering going to West Africa who had not yet volunteered, the most important “enabling” factor, which would make them more likely to go was more information and training (Fig. 1). The availability of effective treatments and/or a vaccine against Ebola also featured highly as factors that would reassure the survey respondents. Interestingly, reassurance about repatriation in the event of contracting Ebola did not feature highly as a barrier, except in the small group of respondents who had actually been to work in the epidemic, where it ranked as the number one concern.

**Fig 2:**
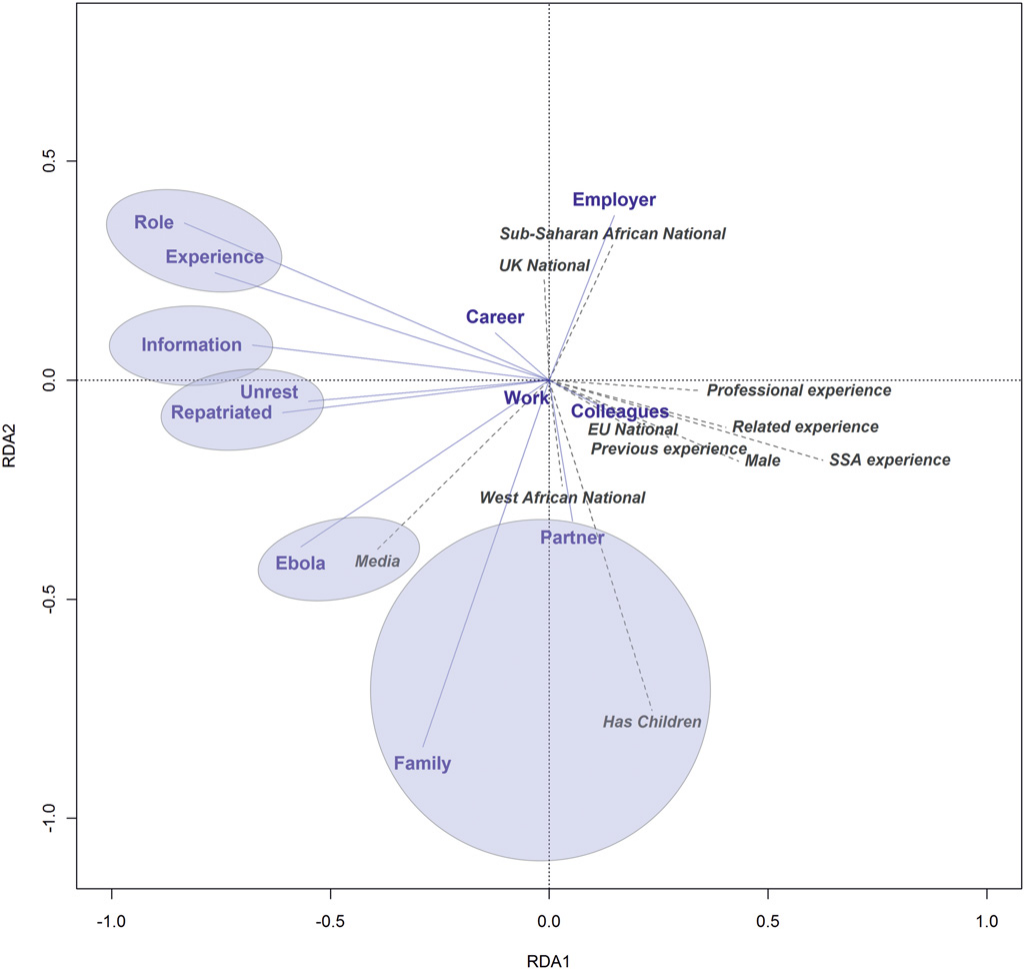
Triplot showing the relationships between barriers and explanatory variables for the group of health workers who are considering going to West Africa, but have not volunteered. Solid blue lines represent barriers; dashed grey lines show explanatory variables. Angles between variables represent their correlations. All the explanatory variables shown have a significant influence on the barriers. RDA 1 and 2 refers to the first two redundancy analysis axes. Circles indicate variables which remained closely correlated across all redundancy analysis axes (equivalent to rotating this figure through different dimensions) that represent significant variation in the dataset (see S1 Fig. for plots of the 2^nd^ and 3^rd^ redundancy analysis axes). SSA experience = experience working in sub-Saharan Africa; previous experience = previous experience of Ebola; related experience = experience of a related transmissible haemorrhagic fever but not Ebola; professional experience = increasing years of experience since primary healthcare qualification. N = 321 respondents from both versions of the questionnaire. Analyses of the additional barriers added in the 2^nd^ version were conducted separately, but did not alter the correlations highlighted (data not shown).

To determine how demographic and other factors influenced the barriers, and whether any particular factors clustered together, we performed redundancy analyses, both on the whole dataset and for the three main groups: *Considering*, *Not Considered*, and *Decided Against*. For this analysis the responses to the question regarding barriers to going were the independent variables, and the demographic (or other) data were the explanatory variables (e.g. age, previous experience, profession, specialty). All variables that showed a significant association with responses regarding barriers are shown on the redundancy analysis plots, Fig. 2 and S1 Fig.

The demographic characteristics for the *Considering* group were very diverse, but in general, those variables that might be expected to cluster together did so. For example, having children was closely associated with having family commitments and reporting a partner’s concerns as barriers. In this analysis lack of sufficient information, which was the most important barrier, was not strongly associated with any other variable, but was loosely associated with two other clusters of barriers: fears of unrest, and worries about not being repatriated if unwell with Ebola and concerns about what the role would be, and insufficient experience and fear of catching Ebola. These factors were less of a concern for the subgroup of people who were considering going and already had experience of working in sub-Saharan Africa; such healthcare workers were more likely to be male and to have longer professional experience.

Across all respondents to the survey, concerns about leaving families or partners were more frequently reported by people with children, and particularly in the 36–45 age group. People with previous experience in sub-Saharan Africa were less concerned that they did not know what they would actually do, or that they did not have the right experience; they were also less concerned about civil unrest or not being repatriated. People who reported that they obtained most of their information about the Ebola crisis through the media were more likely to feel they did not have enough information to inform their decision, and had less idea of what would be expected of them. Doctors with increasing professional experience were most concerned about the impact on their colleagues and families. The younger age groups, and those in allied health professions (pharmacists, biomedical scientists, paramedics and nurses) had more concerns that their career would be adversely affected, or reported that their employer would not allow them to go. These categories of respondents also tended to get more of their information from the media. Those working in infection specialties were the least likely to report having insufficient information.

## Discussion

In this study, 15% of more than 3000 healthcare workers who responded said they are considering going to West Africa to help with the Ebola outbreak; the primary reason why they had not yet volunteered was a lack of information to help them decide. In addition, concerns about what their role would be, and the attitudes of their employer also contributed. All of these are factors could potentially be modified. Fear of contracting Ebola also featured highly among the reasons for not yet volunteering, as well as the concerns of a partner. Our redundancy analyses indicated that, as a barrier, lack of information did not cluster tightly with any of the other barriers, though it did associate loosely with respondents’ concerns about what their role would be, and having insufficient experience, fears of civil unrest, of catching Ebola and of not being repatriated if unwell with the disease. Many of these concerns would likely be allayed with appropriate information, underscoring the importance of this one factor. The absence of adequate information may leave health workers getting more information from the media, which we have shown is associated with greater fear of contracting Ebola compared with obtaining information from more definitive sources such as the medical literature.

Nearly 85% of respondents had either not considered, or had decided against going to help in the epidemic. The overwhelming reasons for this were a partner’s concerns, and family commitments, especially having children or other dependents. Interestingly the responses of those two groups were very similar, suggesting that even those who reported they had not considered going, may have considered it at some level, and decided not to pursue it. Compared with these two groups, those considering going to West Africa were less likely to have children or dependents, and generally perceived all of the barriers to be less of a hindrance, except for lack of access to information.

Because lack of information seemed to be a key factor, we examined the websites of the main organisations who are sending volunteers to work in the Ebola epidemic (The British Red Cross, International Medical Corps, Médecins Sans Frontières, Save the Children, and UK-Med) to see what information was available (Table 3). In general, these organisations are calling for doctors and nurses who have a reasonable amount of experience (over 3 years post registration), preferably including experience in low resource settings. The desirable specialties were emergency medicine, infectious diseases, critical care and paediatrics.

**Table 3:** Current requirements indicated on the websites for organisations sending volunteers to work in the Ebola outbreak in West Africa.

	British Red Cross	International Medical Corps	Médecins sans Frontières
**Experience in resource poor setting**	Yes (desirable)	1–2 years	Yes (3 months working or travelling)
**Clinical experience required—specialty**	Infectious diseases, emergency medicine, anaesthesia, paediatrics, intensive care	Infectious diseases background preferable, Masters in public health preferable	Infectious Diseases
**Clinical experience required—years**	“Extensive”	Minimum 3 years	ST2 or above* (flexible)
**Formal qualification in tropical medicine**	Yes (or experience in tropical medicine)	Desirable	Yes
**Payment/benefits**	Salary, flights, insurance, vaccinations, anti-malarials, bonuses	Vaccinations, insurance	
**Pension**	If secondment arranged can continue contributions	Not mentioned	Not mentioned
**Training**	3 days and further ‘in country’ training	5 days	Exact duration not specified
**Deployment duration**	4 weeks	6 weeks minimum	6 weeks (4 weeks in field)
**Insurance**	Details provided at training	Health insurance and medical emergency insurance provided	Not mentioned
**Post deployment**	Debrief, psychological support, medical check, support for self-monitoring	Not mentioned	Debrief
**Other requirements**	Experience of working in epidemics	Previous NGO experience	Full GMC registration
**How to apply**	E-mail CV and covering letter	Online	Online

(The websites for Save the Children and UK-Med also indicate that they are recruiting volunteers, but we could find no indication of their requirements [24–28]).

Another issue highlighted by those who were considering going was the need for training specific to the tasks that will be carried out. Many potential volunteers seemed unaware that rigorous training is included in the typical 4–6 week deployment. This again highlights the provision of reliable information as a key missing component of the current response. The redundancy analyses for all the groups of respondents showed a strong relationship between reporting there was insufficient information, and obtaining most of their information from the media. Some respondents commented on the fact that if they were asked directly to help out (e.g. via an e-mail), they would be more likely to consider it, rather than just hearing about the need for volunteers via the media. Additional barriers identified by our respondents in free text comments were uncertainty about whether pay would continue as normal and lack of clarity about whether employers would release NHS staff members.

A limitation of our study is that it used a convenience sample and is unlikely to be completely representative of all UK healthcare workers. Given the fast moving nature of the Ebola epidemic the time necessary to fully assess response bias in the UK health worker population would preclude the study results being sufficiently useful to inform policy. A total of 3109 people completed the survey, equivalent to 0.33% of the 937,000 registered doctors, nurses and midwives in the UK [29,30]. Thirty-one percent of respondents worked in acute care specialties, 68% were doctors and 22% were nurses. These figures do not reflect the UK health care worker population, in which nurses outnumber doctors more than two to one. This is perhaps the most significant limitation of our study, as there is a greater need for skilled nursing care than medical expertise in the epidemic. It is possible that this imbalance in respondents has led to us wrongly identifying significant barriers. For example, nurses who had decided against going to work in the epidemic were more likely to cite their employer as a reason not to go than doctors were. However, it is of note that, in the *Undecided* group, there were no significant differences between the nurses responding and any other group. Two percent of our respondents have either made definite plans to go to West Africa, or have actually been and worked in the epidemic. Generalised across the whole health worker population, this would equate to nearly 19,000 volunteers. In reality, the number is nearer 1000. This confirms the expected bias in our responding population in favour of people who are more interested in helping with the epidemic. However, given that this is primarily the group of interest, particularly the barriers and enablers to them going to West Africa, we do not think this is a major limitation. A further limitation is the fact we changed the questionnaire during the study. Nevertheless, the central conclusion of our study, that lack of information is hindering potential volunteers, is based on questions that were present in all versions of the questionnaire and was not influenced by the alteration a week into the survey period.

A final limitation is that we did not specifically ask if the destination country would influence willingness to volunteer. The majority of British health workers volunteering in the Ebola epidemic are being deployed to Sierra Leone because of Britain’s historic links with that country. We detected no evidence from free-text comments that the country of destination would influence the decision to volunteer.

In summary, our study has shown that many more people are considering going to West Africa than have actually signed up, and one of the major factors holding them back is lack of information. Policies which were aimed specifically at addressing this, such as a well-publicised, high quality portal of reliable information, would likely result in more UK healthcare workers volunteering to help tackle Ebola in West Africa.

## Supporting Information

S1 Dataset Survey data. (XLSX)

S1 Fig Triplots for the 2^nd^ and 3^rd^ redundancy analysis axes showing the relationships between barriers and explanatory variables for *“Considering”* health workers. Solid blue lines represent barriers; dashed grey lines show explanatory variables. N = 321. Angles between variables represent their correlations.
(TIF)

S1 File Sources of information and comment regarding potential barriers to volunteering to work in the Ebola epidemic. (DOCX)

S2 File Questionnaire version 1. (DOCX)

S3 File Organisations and other means used to disseminate the questionnaire. (DOCX)

S4 File Questionnaire version 2. (DOCX)

S1 Table Opinions of respondents regarding potential barriers to assisting with the Ebola outbreak in West Africa stratified according to decision made. Data are percentage of respondents to each question.
(PDF)

S2 Table Opinions of respondents regarding potential enablers to assisting with the Ebola outbreak in West Africa stratified according to decision made. Data are percentage of respondents to each question.
(PDF)
