# Supplementary material for: A Survey of UK Healthcare Workers’ Attitudes on Volunteering to Help with the Ebola Outbreak in West Africa
Source: PLoS One. 2015 Mar 11;10(3):e0120013. doi: 10.1371/journal.pone.0120013 (PMC4356617; doi:10.1371/journal.pone.0120013)
Supplement: S1 File — (DOCX) [file pone.0120013.s003.docx]

**Sources of information and comment regarding potential barriers to volunteering to work in the Ebola epidemic:**

BMJ

Nursing Times

Nursing Standard

Lancet

The Times and Sunday Times

The Guardian

The Independent

The Telegraph

Twitter accounts of the above, and of popular medical commentators including @allysonpollock, @NatureNews&Comment, @TomFielden, @ScienceMediaCentre, @PeterHotz
