# Supplementary material for: A Survey of UK Healthcare Workers’ Attitudes on Volunteering to Help with the Ebola Outbreak in West Africa
Source: PLoS One. 2015 Mar 11;10(3):e0120013. doi: 10.1371/journal.pone.0120013 (PMC4356617; doi:10.1371/journal.pone.0120013)
Supplement: S3 File — (DOCX) [file pone.0120013.s005.docx]

**Organisations and other means used to disseminate the questionnaire:**

British Association of Critical Care Nurses (email)

British Infection Association (email)

British Paediatric Allergy, Immunity & Infection Group (email)

College of Emergency Medicine (email)

Intensive Care Society (email)

Joint Royal College Postgraduate Training Board (email)

Letter in the British medical journal

Letter in the Nursing Standard

Letter in the Nursing Times

Northwest HIV nurses network (email, via personal contact)

North west coast & greater manchester clinical research network nurses (email, via personal contact)

Paediatric Intensive Care Society (email)

Royal College of GPs (blog post)

Royal College of Anaesthesia (posted on web page and Twitter)

Royal College of Physicians of Edinburgh (email)

Society of Acute Medicine (email)

**Organisations that were contacted but, to our knowledge, did not send out the survey or did not respond:**

National HIV nurses association

Postgraduate Deaneries: Northern Ireland

Scotland

Wales

Kent Surrey & Sussex

South West (Peninsula)

Wessex

Thames Valley

South London

North Central East London

North West London

East of England

East Midlands

West Midlands

North East

Yorkshire & Humber

Northwest

Mersey

Infectious Diseases Research Network

Liverpool Medical Institution

Médecins sans Frontières (London office)

Royal College of Nursing

Royal College of Midwives

Royal College of Physicians (London)

Royal College of Physicians & Surgeons of Glasgow

Royal College of Surgeons (London)

Royal College of Surgeons (Edinburgh)
