# Supplementary material for: A Survey of UK Healthcare Workers’ Attitudes on Volunteering to Help with the Ebola Outbreak in West Africa
Source: PLoS One. 2015 Mar 11;10(3):e0120013. doi: 10.1371/journal.pone.0120013 (PMC4356617; doi:10.1371/journal.pone.0120013)
Supplement: S4 File — (DOCX) [file pone.0120013.s006.docx]

**QUESTIONNAIRE VERSION 2**

**UK Healthcare workers’ attitudes to Ebola in West Africa: a survey**

**Introduction**

We would like to invite you to participate in a research survey. Before you decide to take part, it is important for you to understand why the research is being done and what it will involve. Please read the following information carefully and feel free to ask us if you would like to know more .

This survey is being sent to doctors, nurses and other healthcare workers to help us understand their thoughts about helping with the Ebola outbreak in West Africa. The results will help to identify and, therefore, address any modifiable barriers there might be. The questionnaire is anonymous but there is an option at the end to separately submit your email address, if you would like more information. The survey consists of 15 questions and will take you around 5 minutes to complete. Please also forward the link (https://www.surveymonkey.com/s/HPRUebola) to any colleagues who may be willing to complete it. Participation is entirely voluntary but by clicking on the 'next' button below you agree to the use of your anonymised data by the University of Liverpool. You are free to exit the survey at any time but any data collected may still be used. All data are anonymous.

If you have any questions or concerns about the survey please feel free to let us know by contacting Prof Tom Solomon and the team at HPRUemerginginfections@liverpool.ac.uk and we will try to help. If you remain unhappy or have a complaint which you feel you cannot come to us with, then you should contact the Research Governance Officer at ethics@liv.ac.uk.

This research is being conducted by the National Institute for Health Research (NIHR) Health Protection Research Unit (HPRU) in Emerging Infections and Zoonoses which is a collaboration between the University of Liverpool, Liverpool School of Tropical Medicine and Public Health England. It is funded by the NIHR. The results, once analysed, will be published in peer reviewed journals. Thank you for taking part.

Dr Lance Turtle, Dr Fiona McGill, Clare Matata RN, Dr Rob Christley, Prof Tom Solomon

NIHR Health Protection Research Unit in Emerging and Zoonotic Infections www.liv.ac.uk/HPRUemerginginfections

Institute of Infection and Global Health, University of Liverpool (www.liv.ac.uk/infectionandglobalhealth)

**1. Have you considered going to West Africa to help in the current Ebola virus epidemic?**

1. IntroductionYour opinions

Options : I have considered going, and decided not to

I have considered going, but am undecided

I have made definite plans to go

I have been and worked in the current epidemic

I haven't considered going

Comments:

**2. We are interested in the reasons people may, or may not, feel able or willing to go to West Africa to participate in efforts to manage the Ebola outbreak. Thinking about your own circumstances, please indicate the extent to which you agree or disagree with the following** statements (answered on a 5 point scale from Strongly Disagree to Strongly Agree):

Options: I have too much of my own work to do to be able to go

I am unwilling to leave my family

I am worried about contracting Ebola virus

My employer will not allow me to go

I do not have the right experience

My partner/other family member does not want me to go

I am worried about increasing the workload of my colleagues if I go

I am worried about violence or civil unrest in West Africa

I am worried that I would not be brought home if I caught Ebola

I don’t know what I would actually do in West Africa

I have not had all the information I need to consider whether or not to help in the current outbreak

I am worried that spending time in West Africa could negatively impact my career

Healthcare workers from the UK will not make any difference to the current outbreak

Illhealth, pregnancy or advancing age prevent me from going.

Comments:

**3.** **I would be more likely to go to West Africa if:**

Options: Alternative cover arrangements are made to fill my post whilst vacant

I received specific training in personal safety/protective equipment/infection control

I had high quality information regarding what would be required of me

I continued to receive my salary whilst away from my current job

An effective vaccine was available

An effective treatment was available

I had first-hand information from someone who had already been and worked in the current Ebola outbreak

Comments:

**4. How much of your information on Ebola virus has come from the following sources:**

Options: Media reports

Medical literature

Discussion with friends / colleagues

Specific course/training/lecture

Actual experience

Related experience (with other directly transmittable viral haemorrhagic fevers)

Authoritative website (Public Health England, WHO, CDC, etc.)

**5. What is your profession?**

Possible responses: Doctor

Nurse

Paramedic

Pharmacist

Biomedical Scientist

Doctor (armed forces)

Nurse (armed forces)

Other

**6. How much experience do you have since you obtained your primary healthcare qualification?**

Possible responses: Less than one year

1-5 years

6-10 years

11-15 years

16-25 years

26- 35 years

36-45 years

>45 years

**7. In what NHS region do you work currently?**

Scotland

Northern Ireland and Isle of Man

Wales

North of England

Midlands and East of England

London

South of England

I don’t live in the UK (please specify where).

8. **Which of the following most closely reflects your current specialty?**

Emergency Medicine/Acute Medicine/Intensive Care Medicine/anaesthesia

Infection Specialty

Other general medical specialty (with internal medicine)

Other general medical specialty (without internal medicine)

Community/primary care

Surgical specialty

Paediatric medical specialty

Paediatric infection specialty

Paediatric emergency medicine

Paediatric intensive care/anaesthesia

Paediatric community based

Paediatric surgical specialty

Obstetrics and Gynaecology

Other

**9. If you are a nurse/allied health professional what band are you?**

Options: 1-3

4-7

8+

**10. If you are a doctor what grade are you?**

Options: F1

F2

ST1/CT1

ST2/CT2

ST3/SpR or above

Consultant

GP

Retired

SAS

**11. How many children living at home or other dependents do you have?**

Options: 0

1

2

3

4

5

>5

**12. Where are you from?**

Options: UK

Other EU Country

West Africa

Other Sub Saharan Africa

Other (please specify)

**13. How old are you?**

Options: <20

20-25

26-35

36-45

46-65

>65

**14. What is your gender?**

Options: Male

Female

**15. Have you ever lived abroad** (options for each answer: no, yes for less than 3 months, yes for between 3 months and a year, yes for longer than a year)?

Options: In Subsaharan Africa

In the tropics other than SubSaharan Africa

Elsewhere

Please use the space below to add any additional comments including your own thoughts about participating in the Ebola outbreak and the factors that may contribute to any decision you have made.
