# Supplementary material for: A Survey of UK Healthcare Workers’ Attitudes on Volunteering to Help with the Ebola Outbreak in West Africa
Source: PLoS One. 2015 Mar 11;10(3):e0120013. doi: 10.1371/journal.pone.0120013 (PMC4356617; doi:10.1371/journal.pone.0120013)
Supplement: S1 Table — Data are percentage of respondents to each question. (PDF) [file pone.0120013.s007.pdf]

Table S1. Opinions of respondents regarding potential barriers to assisting with the Ebola outbreak in West Africa stratified according to decision made. Data are percentage of respondents to each question.

SA = Strongly Agree A = Agree N = Neutral D = Disagree SD = Strongly Disagree

| | Considering (n=472) | | | | | Not considered (n=1791) | | | | | Decided Against (n=704) | | | | | Volunteered (n=53) | | | | | Already Been (n=14) | | | | |
|---|---|---|---|---|---|---|---|---|---|---|---|---|---|---|---|---|---|---|---|---|---|---|---|---|---|
| | SA | A | N | D | SD | SA | A | N | D | SD | SA | A | N | D | SD | SA | A | N | D | SD | SA | A | N | D | SD |
| I have too much of my own work to do to be able to go | 8.4 | 32.3 | 25.3 | 23.8 | 10.2 | 31 | 39.5 | 19 | 7.8 | 2.8 | 20.8 | 35.5 | 20 | 17.9 | 5.8 | 0 | 12.2 | 24.5 | 42.9 | 20.4 | 0 | 8.3 | 25 | 50 | 16.7 |
| I am unwilling to leave my family | 3.2 | 26 | 26 | 33.8 | 11 | 52.1 | 27.6 | 10.9 | 7 | 2.3 | 32.9 | 31.5 | 14.7 | 14.8 | 6 | 0 | 22.9 | 14.6 | 39.6 | 22.9 | 8.3 | 8.3 | 0 | 58.3 | 25 |
| I am worried about contracting Ebola virus | 15.4 | 37.6 | 19.3 | 21.3 | 6.3 | 34.6 | 36.7 | 16.4 | 10 | 2.3 | 22.8 | 35.3 | 21.8 | 16.4 | 3.7 | 8.3 | 41.7 | 27.1 | 14.6 | 8.3 | 8.3 | 8.3 | 16.7 | 58.3 | 8.3 |
| I do not have the right experience | 7.5 | 33.1 | 21.1 | 29.9 | 8.4 | 24.6 | 37.6 | 18.3 | 16.6 | 2.9 | 13.3 | 29 | 20.1 | 29.9 | 7.8 | 4.2 | 2.1 | 20.8 | 50 | 22.9 | 8.3 | 0 | 0 | 50 | 41.7 |
| My employer will not allow me to go | 10.5 | 25.1 | 34.9 | 17.9 | 11.6 | 13.1 | 19.5 | 52.8 | 10.4 | 4.1 | 13 | 18.7 | 41.4 | 19 | 7.8 | 8.3 | 8.3 | 14.6 | 33.3 | 35.4 | 8.3 | 8.3 | 25 | 33.3 | 25 |
| My partner/other family member does not want me to go | 14 | 33.9 | 24.8 | 18.1 | 9.1 | 38.1 | 29.2 | 23.8 | 6.5 | 2.3 | 34.4 | 33.2 | 19.5 | 9.4 | 3.5 | 4.2 | 16.7 | 25 | 33.3 | 20.8 | 0 | 8.3 | 8.3 | 50 | 33.3 |
| I am worried about increasing the workload of my colleagues if I go | 8.2 | 31.4 | 14.3 | 27.9 | 18.2 | 15 | 31.4 | 29 | 17.4 | 7.2 | 12.3 | 29.4 | 22.3 | 25.2 | 10.9 | 0 | 18.8 | 12.5 | 37.5 | 31.2 | 0 | 25 | 16.7 | 50 | 8.3 |
| I am worried about violence or civil unrest in West Africa | 3.9 | 17 | 23.3 | 37.8 | 18 | 14 | 32.7 | 25.3 | 23 | 5 | 6.7 | 21 | 24.6 | 34.7 | 13 | 10.4 | 12.5 | 22.9 | 31.2 | 22.9 | 0 | 16.7 | 8.3 | 50 | 25 |
| I am worried that I would not be brought home if I caught Ebola | 9.3 | 20.3 | 16.2 | 38.4 | 15.8 | 13.8 | 22.9 | 21.7 | 31.8 | 9.8 | 8.4 | 19.6 | 18.3 | 35.9 | 17.8 | 12.5 | 18.8 | 20.8 | 25 | 22.9 | 33.3 | 16.7 | 8.3 | 25 | 16.7 |
| I don't know what I would actually do in West Africa | 7.4 | 37.6 | 16.1 | 31.1 | 7.8 | 17.3 | 42.3 | 19.9 | 16.2 | 4.3 | 8.2 | 33.8 | 17.9 | 29.1 | 11 | 2.1 | 6.2 | 16.7 | 37.5 | 37.5 | 0 | 8.3 | 0 | 41.7 | 50 |
| I have not had all the information I need to consider whether or not to help in the current outbreak | 18.3 | 45.4 | 13.1 | 16.1 | 7.1 | 15.8 | 37.1 | 27 | 14.7 | 5.5 | 9.6 | 33.8 | 24.5 | 23.3 | 8.8 | 2.1 | 12.8 | 17 | 38.3 | 29.8 | 0 | 8.3 | 0 | 50 | 41.7 |
| I am worried that spending time in West Africa could negatively impact my career | 4.3 | 6.9 | 7.6 | 40.6 | 40.6 | 5.4 | 8.2 | 19.7 | 40.9 | 25.8 | 3.8 | 6.7 | 12 | 39.2 | 38.4 | 0 | 10.6 | 2.1 | 27.7 | 59.6 | 0 | 8.3 | 0 | 8.3 | 83.3 |
| Healthcare workers from the UK will not make any difference to the current outbreak | 0.8 | 0.8 | 12.7 | 43.2 | 42.4 | 2.3 | 5.2 | 17.8 | 47 | 27.7 | 2.3 | 4.6 | 9.6 | 40 | 43.5 | 0 | 4 | 4 | 20 | 72 | 0 | 0 | 0 | 25 | 75 |
| Illhealth, pregnancy or advancing age prevent me from going. | 1.7 | 5.1 | 7.6 | 30.1 | 55.5 | 10.3 | 6.5 | 12.3 | 30.5 | 40.5 | 6.8 | 7.4 | 10.9 | 26.8 | 48.2 | 0 | 4 | 16 | 24 | 56 | 0 | 0 | 0 | 25 | 75 |
