# Supplementary material for: A Survey of UK Healthcare Workers’ Attitudes on Volunteering to Help with the Ebola Outbreak in West Africa
Source: PLoS One. 2015 Mar 11;10(3):e0120013. doi: 10.1371/journal.pone.0120013 (PMC4356617; doi:10.1371/journal.pone.0120013)
Supplement: S2 Table — Data are percentage of respondents to each question. (PDF) [file pone.0120013.s008.pdf]

Table S2. Opinions of respondents regarding potential enablers to assisting with the Ebola outbreak in West Africa stratified according to decision made. Data are percentage of respondents to each question.

SA = Strongly Agree A = Agree N = Neutral D = Disagree SD = Strongly Disagree

	Considering (n=472)					Not considered (n=1791)					Decided Against (n=704)					Volunteered (n=53)					Already Been (n=14)				
	SA	A	N	D	SD	SA	A	N	D	SD	SA	A	N	D	SD	SA	A	N	D	SD	SA	A	N	D	SD
**Alternative cover arrangements are made to fill my post whilst vacant**	20.4	35.6	21.9	13	9.1	6.6	26.2	25.8	27	14.4	13.7	34.1	21.9	19.8	10.5	14.6	41.7	20.8	8.3	14.6	25	41.7	16.7	8.3	8.3
**I received specific training in personal safety/protective equipment/infection control**	43.9	41.7	7.5	4.9	1.9	22.3	32.7	15.4	18.5	11	28.6	39.3	15.1	13.2	3.9	38.8	49	6.1	2	4.1	16.7	50	25	0	8.3
**I had high quality information regarding what would be required of me**	47.3	41.5	5.8	3.2	2.2	24.5	34.4	14.9	15.8	10.4	26	46.8	11.8	12.1	3.3	20.4	63.3	8.2	4.1	4.1	16.7	50	16.7	8.3	8.3
**I continued to receive my salary whilst away from my current job**	34.3	33.9	18.5	6.9	6.4	26.8	38.3	15.8	11.7	7.4	27.5	39.8	18.3	8.9	5.4	20.8	41.7	16.7	8.3	12.5	12.5	50	12.5	25	0
**An effective vaccine was available**	39.1	20.4	20	17.4	3	36.5	33.2	14.7	10.3	5.3	38	30	20.5	7.8	3.7	28	36	24	0	12	37.5	25	37.5	0	0
**An effective treatment was available**	35.2	25.8	19.3	16.3	3.4	36.3	34.7	13.7	9.8	5.4	37.2	32.6	19	7.2	4	16	56	16	4	8	37.5	25	37.5	0	0
**I had first-hand information from someone who had already been and worked in the current Ebola outbreak**	22.4	45.3	19.4	11.6	1.3	12.5	33.2	27.9	17.3	9	13.8	41.1	26.4	13.8	4.9	12	48	28	0	12	0	50	37.5	12.5	0
